# Reprocessing of single-use medical devices in cardiology: a systematic literature review of safety and performance characteristics applied to cardiac electrophysiology

**DOI:** 10.1093/europace/euaf316

**Published:** 2026-01-28

**Authors:** Samantha Huynh, Fiona Murphy, Brigitte Sabatier, Judith Pineau, Fabrice Extramiana, Estelle Gandjbakhch, Nicolas Lellouche, Tess Martin, Eloi Marijon, Nicolas Martelli

**Affiliations:** Department of Pharmacy, European Georges Pompidou Hospital, AP-HP, Paris, France; Department of Pharmacy, European Georges Pompidou Hospital, AP-HP, Paris, France; School of Pharmacy and Pharmaceutical Sciences, Trinity College Dublin, Dublin, Ireland; Department of Pharmacy, European Georges Pompidou Hospital, AP-HP, Paris, France; Department of Pharmacy, European Georges Pompidou Hospital, AP-HP, Paris, France; Groupe Parisien Universitaire de Rythmologie (GPUR/AP-HP), Paris, France; Department of Cardiology, Bichat Hospital, AP-HP, Paris, France; Paris-Cité University, Paris, France; Groupe Parisien Universitaire de Rythmologie (GPUR/AP-HP), Paris, France; Department of Cardiology, La Pitié Salpêtrière Hospital, AP-HP, Paris, France; Paris Sorbonne University, Paris, France; Groupe Parisien Universitaire de Rythmologie (GPUR/AP-HP), Paris, France; Department of Cardiology, Henri Mondor Hospital, AP-HP, Créteil, France; Paris-Est-Créteil-Val-de-Marne University, Créteil, France; Department of Pharmacy, European Georges Pompidou Hospital, AP-HP, Paris, France; Paris-Saclay University, GRADES, Orsay, France; Groupe Parisien Universitaire de Rythmologie (GPUR/AP-HP), Paris, France; Paris-Cité University, Paris, France; Division of Cardiology, European Georges Pompidou Hospital, AP-HP, Paris, France; PARCC, INSERM U970, Paris, France; Department of Pharmacy, European Georges Pompidou Hospital, AP-HP, Paris, France; Paris-Saclay University, GRADES, Orsay, France

**Keywords:** Safety, Performance, Reprocessing, Single-use medical device, Electrophysiology catheters, Catheter ablation, Atrial fibrillation, Medical Device Regulation

## Abstract

**Aims:**

The rising burden of cardiovascular diseases, especially atrial fibrillation, has increased demand for electrophysiology (EP) procedures in Europe, driving greater reliance on disposable devices like diagnostic and ablation catheters. Reprocessing single-use medical devices offers potential economic and environmental benefits, yet concerns persist regarding device integrity and safety. Under the European Union Medical Device Regulation, reprocessing is permitted if national laws allow it; however, implementation varies across Member States. This systematic literature review evaluates the safety and performance of reprocessed cardiac EP catheters originally intended for single use.

**Methods and results:**

Following PRISMA guidelines, literature searches of PubMed and Embase identified *in vitro* and *in vivo* studies that examined the safety and functionality of reprocessed EP catheters. Key outcomes included infection risk, device sterility, mechanical and electrical integrity, and adverse events. Twelve studies (four *in vivo* and eight *in vitro*) involving >1200 patients and multiple catheter brands were included. Under stringent reprocessing protocols, reprocessed EP catheters showed comparable safety and mechanical performance to new devices. However, gaps remain in evidence regarding prion and fungal contamination, the maximum number of safe reprocessing cycles, and the detection of rare complications.

**Conclusion:**

The absence of adverse events and reliable performance associated with reprocessed EP catheters reported in this study may encourage European countries that have not yet authorized single-use medical device reprocessing to consider its adoption. However, the broader implementation of this practice remains contingent on its applicability and logistical feasibility within each national context.

## Graphical Abstract

**Graphical Abstract:**
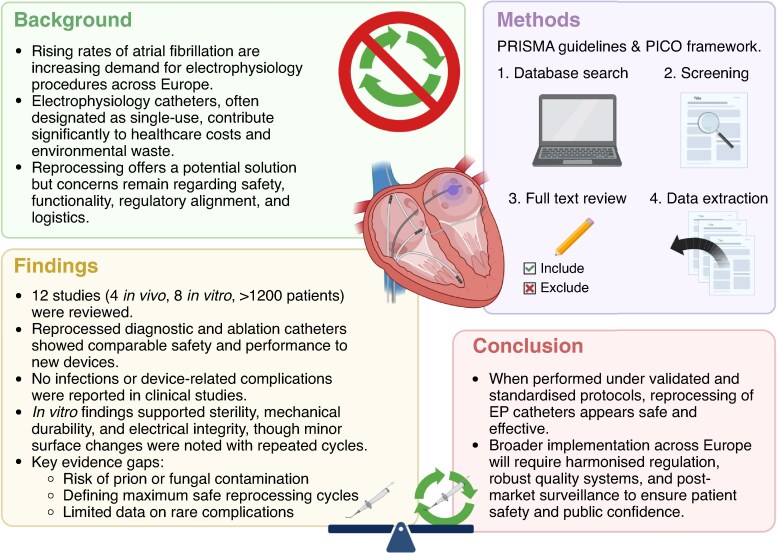

## Introduction

Among the growing challenges in European cardiology in the 21st century is the rise in arrhythmias, namely atrial fibrillation (AF), largely driven by the increasing population, improved survival from other cardiovascular diseases, and the aging population.^1^ Earlier projections suggested that the prevalence of AF in the European Union could exceed 17.9 million by 2060—potentially more than doubling 2010 levels—though estimates may have shifted following the UK’s departure from the European Union.^2^ Consequently, electrophysiological procedures are growing annually by 10–15%, propelled by advancements in diagnostic and interventional technology.^3^

Many electrophysiology (EP) procedures rely on sophisticated single-use medical devices (SUMDs). These devices, though essential, incur substantial costs and exert significant environmental impacts, further straining an already challenged supply chain.^4,5^ Accounting for the majority of SUMD-related environmental impact in cardiology is the use of disposable EP catheters in part due to the use of rare metals such as platinum required for electrical conductivity. Consequently, reprocessing cardiac EP catheters intended for single use has emerged as a viable solution to addressing both the economic and environmental concerns, two highly relevant topics that are discussed extensively elsewhere.^6–8^ Nevertheless, safety and performance concerns with the practice persist.

Reprocessing and further use of CE (*Conformité Européenne*)-marked SUMDs within the European Economic Area (EEA) is permissible under Article 17 of the Medical Device Regulation (MDR), provided national legislation allows it. Reprocessing, as defined in the MDR, includes cleaning, disinfection, sterilization, testing, and restoration to ensure a device’s technical and functional safety and is only permitted if supported by the latest scientific evidence.^9^ Though intended to harmonize medical device legislation, the implementation of Article 17 varies considerably across Member States. Differing national policies on SUMD reprocessing under MDR results in a complex regulatory landscape across the EEA and limits potential cost savings for several Member States’ healthcare systems.^10^ In countries where reprocessing is strictly prohibited,^11^ many cardiologists advocate for a more permissive approach, aligned with that of other EEA countries such as Belgium, Germany, and the Netherlands.^4^

Owing to the lack of a universally applicable methodology to record SUMD-related incidents, safety data primarily rely on literature findings.^10^ Although several isolated studies have demonstrated the safety of reprocessed EP materials intended for single use,^12–14^ Member States have failed to establish a consensus on the practice. Indeed, a 2024 European Commission report highlighted stakeholder concerns regarding potential health risks associated with reprocessed SUMDs, reflecting divergent perceptions of available evidence. The primary safety concern is infection risk, although compromised equipment performance resulting from reprocessing may also compromise safety. Concerns regarding reprocessing of EP catheters also persist among healthcare professionals. Indeed, a 2020 survey of European cardiac electrophysiologists found most respondents perceive the practice as generally safe but expressed concerns about quality aspects (58%), contamination (52%), and loss of precision (47%).^15,16^

Thus, this study examines whether reprocessed cardiac EP catheters intended for single use are safe and effective in light of existing evidence. The aim is to provide an up-to-date systematic literature review evaluating the safety and performance of reprocessed cardiac EP catheters to support informed decision-making regarding the expansion of reprocessing of SUMDs across Member States.

## Methods

### Criteria for considering studies for the review

The Preferred Reporting Items for Systematic Reviews and Meta-Analyses (PRISMA) guidelines were followed.^17^ Data concerning the safety and functionality of reprocessed EP catheters come from both *in vitro* and *in vivo* studies. Although *in vitro* results cannot be directly extrapolated to clinical outcomes, they provide foundational insights into the technical integrity of reprocessed devices and allow for a structured comparison with clinical data. Consequently, two different frameworks were used to inform study selection; a traditional Population, Intervention, Comparison, and Outcome (PICO) framework for the *in vivo* studies and an in-house adapted version for the *in vitro* studies. Given the limited number of *in vivo* studies, *in vitro* data were also analysed to assess whether safety and performance characteristics were consistently reported across study types.

#### PICO framework for *in vivo* studies

The population of interest is patients undergoing cardiac EP procedures that involve the use of cardiac EP catheters. The intervention being evaluated is the use of reprocessed cardiac EP catheters intended for single use only. The comparison group may include cardiac EP catheters that are used as intended for single use only, without undergoing reprocessing or refurbishment. The outcomes of interest are safety and functionality/performance characteristics of the reprocessed cardiac EP catheters. This may encompass measures such as incidence of adverse events related to catheter use, efficacy in delivering intended therapy, or diagnostic capabilities.

#### Adapted PICO framework for *in vitro* studies

The medical devices of interest are cardiac EP catheters intended for single use only, undergoing testing in *in vitro* studies. Topics being evaluated include various reprocessing methods of cardiac EP catheters. These protocols may involve different stages such as cleaning, disinfection, sterilization, and refurbishment to ensure that the catheters can be safely used. A comparison may be made between non-reprocessed cardiac EP catheters intended for single use only (new, unused catheters) and reprocessed catheters. Additionally, comparisons may involve catheters subjected to simulated reuse cycles, assessing the effects of multiple reprocessing rounds on catheter integrity and performance. The outcomes of interest are safety and functionality/performance characteristics of the reprocessed cardiac EP catheters. This includes measures such as tests of sterilization efficacy, validation, mechanical integrity, and usability.

#### Decision-making process for document inclusion and exclusion

The decision-making process for document inclusion followed a structured approach: articles that met the inclusion criteria were included; articles that did not fully meet the criteria (e.g. wrong population or wrong study design) but provided useful background information or could contribute to reflective discussions on the topic were categorized as ‘qualified exclude’; and any documents that neither met the criteria nor were relevant to the scope of the review were excluded. For more details on the inclusion and exclusion criteria, refer to the supplementary material, File S1.

### Search strategy and study selection

Systematic search terms (see Study Protocol in Supplementary material online, *File S1*) were created, and data extracts were retrieved from PubMed and Embase. The extracts from PubMed and Embase were then imported into Rayyan. Rayyan is a web-based software tool designed to streamline the process of conducting systematic reviews and meta-analyses. The software facilitates collaboration among researchers by allowing them to independently screen studies and abstracts and extract data, all within a centralized platform.^18^

Two researchers (F.M. and S.H.) independently and blindly reviewed the titles and abstracts of all articles from the first search. Subsequently, the same two researchers independently reviewed the full texts of the articles deemed potentially suitable for inclusion. Following this, the blind option was lifted, and conflicts were discussed until a consensus was obtained. In case of conflict, the third reviewer (N.M.) had the final say. Subsequently, the snowballing method was used. This involved reviewing the reference lists of included studies and relevant reviews to identify additional studies that met the inclusion criteria.

### Data synthesis

An extraction table was created in Microsoft® Excel for Mac Version 16.85 (24051214) to standardize extraction and analysis of the data (Supplementary material online, File S2). Collected information included details of the first author, journal, year of publication, location, language, catheters used, and safety (sterilization method, retreatment cycles, microbial killing evidence, and validation) and performance characteristics (mechanical integrity and usability).

## Results

### Study selection

**Figure 1:**
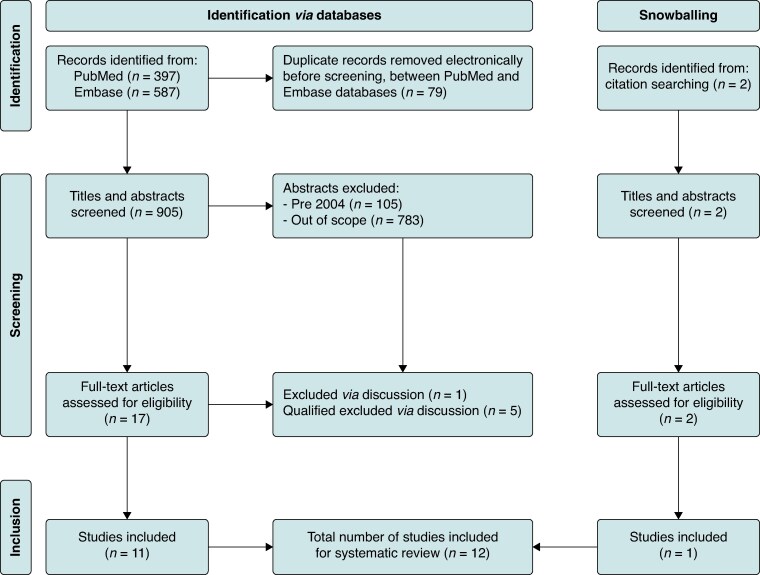
The PRISMA flow diagram for study selection. PRISMA, Preferred Reporting Items for Systematic Reviews and Meta-Analyses.

As shown in *Figure 1*, 905 studies were identified after excluding duplicates. Of these, 888 were excluded based on their titles and abstracts. The remaining 17 studies were reviewed in full, resulting in the exclusion of six more studies. Of these six, five were deemed qualified excludes, e.g. a survey of European electrophysiologists evaluating perceptions of certain sustainable practices in EP including device reprocessing,^6^ while one was excluded. Additionally, two articles were identified via the snowballing method, of which one met the eligibility criteria. Thus, a total of 12 studies were suitable for complete analysis (*Figure 1*)

### Characteristics of included studies

The characteristics of the included studies are summarized in *Table 1*. Of the included studies, seven were conducted in Europe, two in Australia/New Zealand, two in the USA, and one in Brazil. The majority (*n* = 11) were single-centre, with only the New Zealand-based study being multi-centre. Four studies were published from 2019 onwards, while the remaining eight were from 2004 to 2009.

**Table 1:** Characteristics of included studies

Author (year) and location	Title	Study type	Reprocessed and comparison devices	Reprocessing intervention			
				Source of contamination	Decontamination and cleaning process	Sterilization	No. of cycles
Velagic *et al*. (2023)^19^ Croatia	Feasibility and safety of reprocessing of intracardiac echocardiography catheters for EP*^a^* procedures—a large single center experience	Retrospective cohort (*in vivo*) (*n* = 1128)	Intracardiac echocardiography catheters (*n* = 57) None	Clinical use	Not specified	Hydrogen peroxide gas plasma	Up to 20
Wilson *et al*. (2020)^20^ New Zealand	Initial experience with a novel re-sterilisable decapolar EP catheter	Case–control (mixed) (*n* = 64)	Catheter handles (*n* = 11) None	Clinical use	Not specified	Autoclaving	Up to 12
Leichsenring *et al*. (2018)^21^ Brazil	Conception and validation of a protocol for reuse of non-irrigated EP catheters in a Brazilian teaching hospital	Validation (mixed) (*n* = 75)	EP catheters (*n* = 68) None	Clinical use	Mechanical cleaning using enzymatic solution, ultrasonication, and hydrogen peroxide	Ethylene oxide	Six to nine
Leung *et al*. (2019)^22^ UK	Remanufactured circular mapping catheters: safety, effectiveness and cost	Case–control (*in vivo*) (*n* = 100)	Circular mapping catheters (*n* = not specified) New catheters	Clinical use	Not specified	Ethylene oxide	Not specified
Lester *et al*. (2009)^23^ USA	Reprocessing and sterilization of single-use electrophysiological catheters: removal of organic carbon and protein surface residues	Experimental (*in vitro*)	EP catheters (*n* = 168) New catheters (*n* = 51)	Mixture of clinical use and worst-case soiling and simulated reuse	Multiple	Ethylene oxide	Five
Tessarolo *et al*. (2007)^24^ Italy	Health technology assessment on reprocessing single-use catheters for cardiac EP: results of a three-years study	Experimental (*in vitro*)	EP catheters (*n* = 20) None	One clinical use followed by repeated simulation cycles	Multiple	Unknown	Five
Tessarolo *et al*. (2007)^25^ Italy	Different experimental protocols for decontamination affect the cleaning of medical devices. A preliminary electron microscopy analysis	Experimental (*in vitro*)	EP catheters (*n* = 20) None	One clinical use followed by repeated simulation cycles	As for Tessarolo *et al.* (2007)	Not applicable	Not specified
Tessarolo *et al*. (2006)^26^ Italy	Sterility and microbiological assessment of reused single-use cardiac EP catheters	Experimental (*in vitro*)	EP catheters (*n* = 208) None	One clinical use followed by repeated simulation cycles	Chlorine solution, enzymatic solution, and manual brushing	Hydrogen peroxide gas plasma	Three
Tessarolo *et al*. (2006)^27^ Italy	Efficiency in endotoxin removal by a reprocessing protocol for EP catheters based on hydrogen peroxide plasma sterilization	Experimental (*in vitro*)	EP catheters (*n* = 61) None	Clinical use	Chlorine solution, enzymatic solution, and manual brushing	Hydrogen peroxide gas plasma	Not specified
Lester *et al*. (2006)^28^ USA	Comparison of performance characteristics between new and reprocessed EP catheters	Experimental (*in vitro*)	EP catheters (*n* = 165) New catheters (*n* = 58)	Clinical use (five cycles) and simulated soilage	Enzymatic detergent and ultrasonication	Unknown	Five
Druce *et al*. (2005)^29^ Australia	Cleaning and sterilization protocol for reused cardiac EP catheters inactivates hepatitis and coxsackie viruses	Experimental (*in vitro*)	EP catheters (*n* = 120)	Clinical use (nine cycles) and simulation with infected blood	Detergent treatment with manual brushing then enzymatic solution	Ethylene oxide	Not specified
Tessarolo *et al*. (2004)^30^ Italy	Evaluation and quantification of reprocessing modification in single-use devices in interventional cardiology	Experimental (*in vitro*)	EP catheters (*n* = 7)	Clinical use	Chlorine solution and enzymatic solution	Hydrogen peroxide gas plasma	Not specified

^a^EP: electrophysiology.

Across the twelve included studies, almost all (*n* = 11) focused on diagnostic and ablation catheters intended for single use only. One study examined a novel decapolar catheter designed to be reprocessed with a disposable sheath for each use. The main EP devices examined were diagnostic and ablation catheters, although one study evaluated intracardiac echocardiography catheters.^19^ Of the twelve studies, four included *in vivo* data, collectively involving over 1200 human subjects. The majority of *in vivo* patient data (*n* = 1128) comes from a single large observational study by Velagic *et al*.^19^ The remaining studies (*n* = 8) focused on *in vitro* experiments.

The reprocessing interventions varied among the studies in terms of contamination sources, decontamination and cleaning processes, sterilization methods, and the number of reprocessing cycles. All studies involved at least one clinical use, with some employing simulated reuse protocol(s) to mimic worst-case scenarios. Multiple sterilization methods were used, with ethylene oxide (*n* = 4) and hydrogen peroxide gas plasma (*n* = 4) being the most common. Only one study used traditional autoclaving, and the sterilization method in the three remaining studies was not clearly stated.

The recommended number of reprocessing cycles, where safety and functionality were maintained, varied from 3 to 20 cycles, emphasizing the variability in findings across studies.

### Performance characteristics

A summary of the performance characteristics may be found in *Table 2*. All *in vivo* studies reported reliable mechanical integrity and durability comparable to first-use catheters, with signal quality and communication deemed clinically acceptable for several reprocessing cycles. *In vitro* tests showed more variable results. Some studies found that appropriate reprocessing methods preserved mechanical and electrical integrity, while others identified reprocessing-related damage to EP catheters. Tessarolo *et al.*^25,26^ reported that certain degradation patterns were linked to specific cleaning protocols, with chlorine-based agents causing protein denaturation and aggressive chemicals contributing to material degradation over time. Another study from the same group indicated that micro-scratches and changes in nano-roughness correlated with the number of reprocessing cycles, suggesting potential mechanical integrity concerns.^30^ Nevertheless, studies on electrical integrity did not raise concerns, even after multiple reprocessing cycles.

**Table 2:** Performance characteristics of reprocessed cardiac EP catheters

Author (year)	Mechanical integrity	Electrical/image integrity
***In vivo***		
Velagic *et al*. (2023)^19^	Durable up to 20 cycles; no mechanism malfunction	Minor image decline with reuse; adequate visualization
Wilson *et al*. (2020)^20^	Reused 12 times; 92% coronary sinus placement success	Clinically acceptable pacing and signal quality
Leichsenring *et al*. (2018)^21^	Handling issues led to 1/3 catheters discarded (technical problems, displacement of electrodes, and cleaning difficulties)	Conduction and polymer surface integrity maintained; minor surface damage possible
Leung *et al*. (2019)^22^	Handling comparable to first use; 1% failure rate	Satisfactory electrogram and mapping performance
***In vitro***		
Lester *et al*. (2009)^23^	Residues removed, preserving device integrity	Not explicitly assessed
Tessarolo *et al*. (2007)^24^	Lubrication worsened after four cycles due to surface roughness	No variations in ablation efficiency, conductivity, or sensor accuracy
Tessarolo *et al*. (2007)^25^	Enzymatic and ultrasonic cleaning preserved mechanical properties; aggressive chemicals caused degradation	No detailed data provided
Lester *et al*. (2006)^28^	Reprocessed met industry mechanical standards for integrity	Maintained electrode continuity and isolation
Tessarolo *et al*. (2006)^26^	Integrity maintained; specific degradation patterns linked to cleaning protocols (e.g. chlorine-based cleaning)	No detailed data provided
Tessarolo *et al*. (2006)^27^	Efficiency in endotoxin removal by a reprocessing protocol for EP*^a^* catheters based on hydrogen peroxide plasma sterilization	No detailed data provided
Druce *et al*. (2005)^29^	Mechanical integrity preserved with strict protocols	Thorough cleaning is critical for safety
Tessarolo *et al*. (2004)^30^	Micro-scratches and nano-roughness observed; potential mechanical integrity degradation	Potential impact on function; no detailed data provided

^a^EP: electrophysiology.

### Safety characteristics

A summary of the safety findings is presented in *Table 3*. Of the four *in vivo* studies included, none of the >1200 participants developed any complications, allergies, or infections that could be attributed to the use of reprocessed cardiac EP catheters. Several of the included studies demonstrated that well-defined reprocessing protocols can successfully remove detergent residues and biological contamination as well as achieve sterility. One study demonstrated successful inactivation of blood-borne virus surrogates.^29^ Numerous authors highlight the importance of using filtered water in cleaning protocols instead of potable water to avoid contamination with endotoxins.^24,26,28^ Nevertheless, it appears that any endotoxins introduced may be successfully removed using hydrogen gas plasma sterilization. One study raised concerns about the risks posed by chlorine-based cleaning solutions to healthcare staff and highlighted the toxicity of polyphenolic-based treatments.^25^ The possibility of phenol toxic effects during EP interventions and the need for personal protective equipment were noted. Negative findings included the potential for chemical composition changes in the device’s external molecular layer, which may predispose to thrombus formation and bacterial adhesion. One study highlighted the difficulty in quantifying the risk of blood-borne virus transmission during actual EP procedures.^29^ None of the included studies explicitly addressed fungal or prion contamination.

**Table 3:** Safety characteristics of reprocessed cardiac EP catheters

Author (year)	Risk of infection and microbiological findings	Other risks related to the reprocessing method	Safety conclusions and/or recommendations
***In vivo***			
Velagic *et al*. (2023)^19^	None reported	None reported	Reprocessing of intracardiac echocardiography catheters appears safe. Larger studies are required to identify rarer complications.
Wilson *et al*. (2020)^20^	None reported	None reported	Reprocessing of this partially reusable catheter appears safe.
Leichsenring *et al*. (2018)^21^	None reported	None reported	Reuse of EP^a^ catheters under the conditions used in the study may be considered safe for patients.
Leung *et al*. (2019)^22^	None reported	None reported	Remanufactured circular mapping catheters are safe. Consider the potential for prion disease transmission.
***In vitro***			
Lester *et al*. (2009)^23^	Sterility and acceptable endotoxin content can be realized with specific protocols.	Can successfully remove detergent residues with a well-defined cleaning protocol	Defined reprocessing protocols can effectively remove detergent residues and biological contamination.
Tessarolo *et al*. (2007)^24^	Sterility can be achieved for three reprocessing cycles.	There is a theoretical risk of pyrogenic reactions should potable water be used during the reprocessing cycle.	Non-pyrogenic water should be used during the cleaning process. Recommend a precautionary number of regenerations of five cycles.
Tessarolo *et al*. (2007)^25^	Not discussed	Certain cleaning protocols may present a risk to healthcare staff, e.g. chlorine-based solutions. Polyphenolic-based cleaning treatments are effective but could cause serious toxicity on reuse.	Consider the possibility of phenol toxic effects if released into the bloodstream during intervention. Consider the need for personal protective equipment for staff based on the cleaning protocol.
Lester *et al*. (2006)^28^	The protocol used was insufficient to guarantee sterility after five reuses.	Non-sterile water may introduce Gram-negative bacteria contamination. Unsuitable storage conditions before resterilization hinder the ability of the reprocessing method to demonstrate sterility.	Reprocessing should only be done in hospitals with considerable workloads. Use purified or sterile water and autoclaved non-linting tissues. Optimize storage conditions during the reprocessing cycle.
Tessarolo *et al*. (2006)^26^	Not discussed	Potable water and manual cleaning may introduce endotoxins. The pyrogenic load was satisfactorily reduced following hydrogen peroxide gas plasma treatment.	Hydrogen peroxide gas plasma sterilization is an efficient treatment for non-lumen cardiac EP catheters.
Tessarolo *et al*. (2006)^27^	Not discussed	No significant difference in the comparison of the force to initiate tip buckling between new and processed catheters	Catheters may undergo five use/reprocessing cycles while maintaining safety characteristics consistent with a new device.
Druce *et al*. (2005)^29^	Successful inactivation of blood-borne viruses is possible.	Not discussed	Be aware that it is not possible to quantify the risk of transmission of blood-borne viruses during an actual procedure from this study.
Tessarolo *et al*. (2004)^30^	Increasing surface roughness and chemical variations in the external molecular layer can favour bacterial adhesion.	Increasing surface roughness and variations on chemical composition in the external molecular layer can favour thrombus formation.	The reprocessing method should be individualized to the device.

^a^EP: electrophysiology.

## Discussion

This systematic literature review represents the first comprehensive evaluation of the safety and performance characteristics of reprocessed cardiac EP catheters intended for single use. Our findings suggest that, when reprocessing is conducted under stringent protocols, these catheters demonstrate comparable safety and functionality to new devices.

### Performance

Critical performance attributes such as flexibility, imaging integrity, electrical conductivity, and insulation are particularly sensitive to reprocessing effects, requiring rigorous evaluation to ensure their safety and functionality. Nevertheless, the potential clinical impact of a small proportion of dysfunctional reprocessed EP catheters must be weighed against the broader benefits of reprocessing. Even in such cases, catheter replacement following vascular access is typically rapid and does not meaningfully delay the procedure. Moreover, unlike single-use catheters, which are subject to batch-level quality control, reprocessed catheters undergo individual functional and integrity assessments prior to use. This systematic verification has the potential to enhance overall device reliability and reduce the incidence of catheter-related performance issues in clinical practice.

The reviewed studies generally support that when reprocessing is conducted under stringent protocols, reprocessed EP catheters can maintain functionality comparable to new devices. In practice, this involves thorough cleaning and rinsing of the probe after use, followed by a series of visual, mechanical, and microbiological checks, some of which are defined by ISO standards or protected by patent. However, the extent to which performance is preserved varies, particularly between *in vivo* and *in vitro* studies, highlighting the need for further scrutiny. *In vivo* studies, which assess device function under real-world clinical conditions, consistently found that reprocessed catheters remained mechanically durable, with no major malfunctions reported over multiple cycles. Some research suggested a slight decline in performance metrics such as image quality and handling, yet these changes did not appear to compromise clinical utility. The findings from Velagic *et al.*,^19^ for instance, indicate that reprocessed intracardiac echocardiography catheters retained structural integrity for up to 20 reuses, though minor image degradation was observed. Overall, these results reinforce the argument that reprocessing, if performed correctly, can extend EP catheter usability without significantly affecting function.

In contrast, *in vitro* studies paint a more variable picture. Some research demonstrated that reprocessed catheters met established performance benchmarks, while others raised concerns about structural degradation over repeated cycles. Tessarolo *et al.*^24,30^ identified microscopic surface alterations, including micro-scratches and changes in nano-roughness, which correlated with the number of reprocessing cycles. Although these findings do not necessarily imply immediate clinical risk, they suggest that reprocessed catheters could be more prone to wear over time, particularly with repeated handling and flexion.^22^ Additionally, although chlorine-based detergents may contribute to material degradation, their use is not standard practice in established reprocessing protocols, limiting the relevance of these findings to clinical settings. Overall, the extent to which these microstructural changes translate into functional limitations in clinical settings remains uncertain. Indeed, no studies to date have demonstrated that these microstructural changes directly affect procedural confidence or usability in cardiac EP. Specifically, there is no evidence that operators experience reduced confidence in image integrity or the procedure itself when using reprocessed EP catheters. These results reinforce the need for ongoing monitoring of catheter integrity after multiple reprocessing cycles.

### Safety

Across twelve studies involving >1200 patients, there were no reported infections, allergic reactions, or complications directly attributable to reprocessed EP catheters. Sterility was consistently maintained across multiple reprocessing cycles, though the maximum number of safe reuses varied depending on the method and device, reinforcing the need for validated reprocessing protocols. However, several authors emphasized that the effectiveness of reprocessing is highly dependent on protocol quality, with concerns raised about endotoxin contamination from potable water, highlighting that potable water should be avoided during reprocessing.^24,26,28^

The *in vivo* studies demonstrated no clinical safety concerns with the reprocessed EP catheters, and *in vitro* investigations provided direct microbiological assessments, confirming that reprocessing protocols were capable of achieving sterility. One *in vitro* study specifically demonstrated successful inactivation of blood-borne viruses, further supporting the microbiological safety of reprocessing under controlled conditions.^29^ However, gaps remain in the assessment of other microbiological risks. No study explicitly evaluated the potential for fungal contamination or prion disease transmission, leaving uncertainty regarding the effectiveness of existing reprocessing protocols against these pathogens. Unlike bacteria and viruses, prions lack nucleic acids, making them resistant to conventional sterilization methods such as autoclaving or ethylene oxide gas sterilization. Although no cases of iatrogenic prion transmission have been linked to reprocessed EP catheters, documented cases of prion transmission *via* inadequately sterilized neurosurgical instruments highlight the need for continued vigilance and strict decontamination guidelines. Indeed, isolated case reports in the neurosurgical setting have shown that prions can persist on surgical instruments despite standard sterilization measures, leading to the transmission of Creutzfeldt-Jakob disease.^31^ Though prion disease transmission has been documented following neurosurgical procedures involving direct brain contact, the relevance to EP catheters remains uncertain, as these devices do not come into contact with high-risk neural tissues. Nevertheless, given the resistance of prions to conventional sterilization methods, continued vigilance is warranted when considering reprocessing protocols for any device reused in a clinical setting.^32^ Ahead of broader implementation, future research should focus on developing targeted microbiological validation protocols specifically designed to assess the resistance profiles of fungal pathogens and prions, thereby addressing these remaining evidence gaps.

Reprocessed EP catheters are considered safe under controlled conditions. However, there is a lack of long-term data on the cumulative effects of repeated sterilization on microbiological safety. Further research is needed to assess microbiological risks, optimize decontamination protocols, and establish validated resterilization limits. As European regulatory bodies re-evaluate reprocessing policies, these insights are valuable for understanding the potential benefits and risks associated with expanding reprocessing practices for EP catheters.

### Legal and regulatory considerations

Germany is widely recognized as the European leader in SUMD reprocessing, having authorized the practice under national legislation more than two decades ago preceding the introduction of MDR. The German legal framework supports both open- and closed-loop reprocessing systems as well as hospital-based reprocessing. Nevertheless, Germany has prioritized the establishment of third-party reprocessing infrastructure to successfully manage off-site SUMD reprocessing, reducing variability in cleaning, sterilization, and quality control processes.^33^ Germany’s approach showcases how other EEA Member States may successfully integrate the practice into their own healthcare systems under MDR.^11^

**Figure 2:**
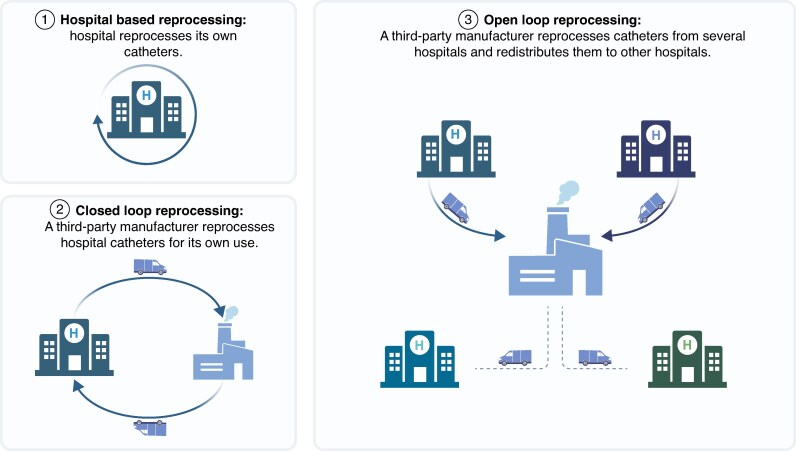
Models for reprocessing EP catheters. EP, electrophysiology.

Importantly, though reprocessing is defined under MDR, the specific systems and operational methods by which SUMDs may be reprocessed are not explicitly described. As shown in *Figure 2*, there are three models for reprocessing EP catheters: (i) hospital-based reprocessing, where a hospital reprocesses its own devices; (ii) closed-loop reprocessing, where a third-party manufacturer reprocesses devices and returns them to the originating hospital; and (iii) open-loop reprocessing, where third-party manufacturers collect devices from multiple hospitals and redistribute reprocessed products across different institutions (*Figure 2*).

Each system carries different implications for regulatory responsibility and clinical accountability. Under the MDR, the reprocessor becomes the new legal manufacturer. The hospital-based reprocessing model may raise significant concerns for cardiologists, who may be reluctant to use reprocessed SUMDs if their institution bears direct clinical and legal responsibility for device quality, especially in high-risk cardiac procedures. Moreover, third-party reprocessors often rely on complex industrial processes, such as ethylene oxide sterilization. Though effective, ethylene oxide is a carcinogenic and toxic agent subject to stringent occupational and environmental safety regulations.^34^ This makes its use infeasible in most hospital settings and contributes to the limited development of hospital-based reprocessing systems, potentially explaining why Germany has largely focused on developing third-party reprocessing infrastructure within an open-loop system.^16^

### Limitations

This review is constrained by the marked heterogeneity of the included studies in terms of design (*in vivo* vs. *in vitro*), types of catheters, reprocessing protocols, and measured outcomes, which makes direct comparisons or pooled quantitative analyses infeasible. Although some research enrolled sizable patient cohorts, *in vivo* data remain relatively scarce and often rely on older protocols that may not accurately reflect current device technology and materials or evolving regulatory requirements. Consequently, these limitations may affect the overall strength of the study and generalizability of our findings.

Additionally, the majority of clinical safety data in this review comes from the study of Velagic *et al.*,^19^ which comprises over 90% of the total patient cohort. Although the size of this dataset is reassuringly large, the findings are based on a specific sterilization protocol and deal with intracardiac echocardiographic catheters only. Thus, these results should be interpreted with caution when considering broader generalizability of the findings to all EP catheter types.

In Europe, where historically the reprocessing of single-use devices has been prohibited and on-site ethylene oxide capacity is limited, there is a large absence of local data, restricting the applicability of our findings. Additionally, a potential limitation of our study is the failure to identify all available data on the topic, partly due to publication bias. The diverse nature of the medical literature on reprocessing, with variations in study design and interventions, further complicates the synthesis of information.

### Future perspectives

Increased environmental scrutiny and economic pressures are likely to drive growing interest in reprocessing across Europe. Although the environmental benefits of reprocessing are frequently cited, they have yet to be conclusively demonstrated and may vary depending on the reprocessing model used. Some studies suggest that reprocessing single-use EP catheters can reduce their carbon footprint by 50 to 60% over the device’s life cycle.^35^ However, these findings are primarily based on open-loop models involving third-party reprocessors and may not be directly generalizable to other systems, such as hospital-based reprocessing. Similarly, a 2024 French government report also estimated that reprocessing could reduce hospital costs by up to 60%.^36^ Since reprocessing EP catheters similarly reduces per-procedure costs while maintaining clinical performance, it represents a concrete value-based healthcare intervention that directly addresses the ‘escalating costs’ of advanced EP technologies highlighted by Osoro *et al.*^37^ As sustainability becomes a more prominent policy objective amid an uncertain economic climate, robust, context-specific data will be essential to guide evidence-based decision-making and support the development of scalable reprocessing infrastructure across Europe.

This review highlights that reprocessed EP catheters intended for single use can maintain safety and functionality under stringent protocols, reinforcing the potential to expand the practice in Europe. While *in vivo* studies support the safety of reprocessed EP catheters, gaps remain regarding prion and fungal contamination, the number of safe reprocessing cycles, and the detection of rare complications. Future vigilance measures, akin to the EudraVigilance system used in pharmacovigilance, could facilitate long-term safety monitoring of reprocessed SUMDs without requiring patient registries. Finally, opponents of reprocessing of SUMDs raise ethical concerns, including prion risks, performance issues, and the expectation of new equipment. Although these are important during the experimental phase, they should not prevent a shift towards a more sustainable model. Highlighting patients’ role in supporting responsible healthcare, alongside transparent communication and thorough safety evaluations, will help build confidence in reprocessing practices and ensure decisions prioritize both patient safety and environmental responsibility.

Despite growing evidence supporting SUMD reprocessing, logistical and regulatory challenges remain key barriers to widespread adoption across Member States. One of the primary obstacles is the limited availability of third-party reprocessing facilities in several EEA countries, restricting access despite demand from high-volume EP centres. A centralized third-party model, as seen in Germany, could help standardize domestic reprocessing in EEA Member States, mitigating the risks associated with hospital-based inconsistencies in decontamination methods and equipment availability. Though other Member States could pursue similar pathways, the lack of specialized expertise and infrastructure may present a significant barrier to implementation. This presents an opportunity for a coordinated European approach, perhaps by the creation of a European hub for standardized SUMD reprocessing, enabling subsequent export and use across all Member States that permit the clinical use of such devices under national legislation.

## Conclusions

The literature indicates that reprocessed cardiac EP catheters, originally intended for single use, demonstrate safety and performance comparable to their single-use counterparts. Although evidence gaps remain, particularly concerning prion and fungal contamination and the logistical feasibility of reprocessing within EEA Member States, the available data support the safety and performance of reprocessed devices. Further evaluation at the European level could help inform regulatory decision-making and support a more harmonized approach to single-use medical device reprocessing.

## Supplementary Material

euaf316_Supplementary_Data

## Data Availability

The data supporting the findings of this study are available on request from the corresponding author.
