# Supplementary material for: Reprocessing of single-use medical devices in cardiology: a systematic literature review of safety and performance characteristics applied to cardiac electrophysiology
Source: Europace. 2026 Jan 28;28(1):euaf316. doi: 10.1093/europace/euaf316 (PMC12849812; doi:10.1093/europace/euaf316)
Supplement: euaf316_Supplementary_Data [file euaf316_supplementary_data.zip › Supplementary material file S1.pdf]

# Aims and Objectives

The aim of this systematic literature review is to describe the safety and performance characteristics of reprocessed cardiac EP catheters intended for single use only.
Specifically, the objectives of this systematic literature review are:

- To evaluate the safety of reprocessed EP catheters.
- To assess the performance characteristics of reprocessed EP catheters.
- To compare the outcomes of reprocessed EP catheters with non-reprocessed, single-use EP catheters.

# Methods

## Criteria for Considering Studies for the Review

The Preferred Reporting Items for Systematic Reviews and Meta-Analyses (PRISMA) guidelines will be used to inform the methodology. As data concerning the safety and functionality of reprocessed EP catheters come from both *in vitro* and *in vivo* studies, a broad approach to the literature search will be applied. The distinction between *in vivo* and *in vitro* studies is crucial due to the inherent differences in their methodologies and focus. *In vivo* studies will evaluate the performance and safety of reprocessed catheters in clinical settings, directly involving patients and assessing clinical outcomes. Conversely, *in vitro* studies will focus on laboratory-based evaluations, testing the technical and functional aspects of reprocessed catheters under controlled conditions. This separation will allow for a comprehensive assessment of both the clinical and technical dimensions of catheter reprocessing, ensuring a thorough understanding of its implications across different contexts. To address these differences, the following PICO frameworks will be used.

### *In Vivo* Studies

The population of interest will be patients undergoing cardiac electrophysiology procedures that involve the use of catheters. This may include individuals with various cardiac conditions requiring diagnostic or therapeutic interventions. The intervention being evaluated will be the use of reprocessed cardiac electrophysiology catheters intended for single use only. This will include catheters that have undergone reprocessing or refurbishment for reuse in cardiac procedures. The comparison group may include cardiac electrophysiology catheters that are used as intended for single-use only, without undergoing reprocessing or refurbishment. A comparison will be drawn between patients who undergo EP catheterisation using reprocessed equipment versus catheters used as intended. The outcomes

of interest will be safety and functionality/performance characteristics of the reprocessed cardiac electrophysiology catheters. This may encompass measures such as incidence of adverse events related to catheter use, efficacy in delivering intended therapy or diagnostic capabilities.

**Adapted PICO Framework for *In Vitro* Studies**

The medical devices of interest will be cardiac electrophysiology catheters intended for single use only, undergoing testing in *in vitro* studies. The protocol being evaluated will include various reprocessing methods of cardiac electrophysiology catheters. These protocols may involve different stages such as cleaning, disinfection, sterilisation, and refurbishment to ensure that the catheters can be safely reused. Cleaning will involve the removal of organic and inorganic materials from the device. Disinfection will reduce the number of viable microorganisms to a safe level. sterilisation will completely eliminate all forms of microbial life. Refurbishment may include restoring the device's technical and functional performance to its original specifications.

A comparison will be made between non-reprocessed cardiac electrophysiology catheters intended for single use only (new, unused catheters) and reprocessed catheters. Additionally, comparisons may involve catheters subjected to simulated reuse cycles, assessing the effects of multiple reprocessing rounds on catheter integrity and performance.

The outcomes of interest will be safety and functionality/performance characteristics of the reprocessed cardiac electrophysiology catheters. This will include measures such as sterility (assessment of microbial contamination and sterilisation efficacy), validation (evidence of microbial killing and effective reprocessing protocols), mechanical integrity (durability and structural integrity after reprocessing), and usability (functional reliability and efficacy in delivering intended therapy or diagnostic capabilities).

## Inclusion and Exclusion Criteria

The inclusion and exclusion criteria may be seen in Table 1 below.

**Table 1.** Inclusion and Exclusion Criteria

| Inclusion Criteria | Exclusion Criteria |
|---|---|
| All non-implantable cardiac EP catheters including mapping, diagnostic, and ablation catheters. | |
| Report on safety and/or performance characteristics of reprocessed catheters intended for single use in vivo or in vitro. Including microbiological assessments and infectious tests. | Devices other than electrophysiological catheters, such as pacemakers, implantable cardioverter-defibrillators or other implantable cardiac devices. |
| From 2004 onwards | Pre 2004 |
| English or French language | Any other language |
| Partially or totally reused catheters | |
| Reports on at least one outcome of interest, i.e., safety or functionality. | |

The decision making-process for document inclusion will be as follows:

- Include: Papers that meet the inclusion criteria.
- Qualified Exclude: Articles that do not completely meet the inclusion criteria but provide useful background information or could contribute to reflective discussions on the topic.
- Exclude: Any document that does not meet the inclusion criteria and is completely outside the scope of the review.

## Search Strategy and Study Selection

Systematic search terms will be created and extracts will be pulled from PubMed, and Embase. The extracts from PubMed and Embase will then be imported into Rayyan. Rayyan is a web-based software tool designed to streamline the process of conducting systematic reviews and meta-analyses. The software facilitates collaboration among researchers by allowing them to independently screen study titles and abstracts and extract data, all within a centralised platform. Rayyan offers features such as automatic de-duplication of references, blind review options to reduce bias, and customizable inclusion and exclusion criteria.

Two researchers (FM, SH) will independently and blindly review the titles and abstracts of all articles from the first search. Subsequently, the same two researchers will independently review the full texts of the articles deemed potentially suitable for inclusion. Following this, the blind option will be lifted and conflicts will be discussed until consensus is obtained. In case of conflict, the third reviewer (NM) will have the final say. Subsequently, the snowballing method will be used. This will involve reviewing the reference lists of included studies and relevant reviews to identify additional studies that meet the inclusion criteria.

## Search Terms

The search terms used may be seen below.

**PUBMED**

((”electrophysiology catheter*”) OR (”EP catheter*”) OR (”cardiology device”) OR (”cardiac device”) OR (”electrophysiology material”) OR (”electrophysiology equipment”) OR (”catheter ablation”) OR (”radiofrequency ablation”) OR (”cryoablation”) OR (”mapping catheter*”) OR (”diagnostic catheter*”) OR ”cardiac electrophysiology” [MeSH Terms]) AND ((”reus*”) OR (”reprocess*”) OR (recycl*) OR (”single-use”) OR (”single use”) OR (”remanufactur*”) OR (”cleaning”) OR (”disinfection”) OR (”autoclaving”) OR (”sterilisation”) OR (”sterilisation”) OR (sterility) OR (”decontamination”) OR (”Equipment Reuse”[MeSH Terms])) AND ((”safe*”) OR (”function*”) OR (”perform*”) OR (”effective*”) OR (”efficacy”) OR (”protocol”) OR (”equivalent*”) OR (”durability”) OR (”reliability”) OR (”sterility assurance”) OR (”clinical outcomes”) OR (”risk assessment”) OR (”quality control”) OR (”infect*”) OR (microbio*) OR (”recommendation*”))

**EMBASE**

(’electrophysiology catheter*’ OR ’ep catheter*’ OR ’cardiology device’ OR ’cardiac device’ OR ’electrophysiology material’ OR ’electrophysiology equipment’ OR ’catheter ablation’ OR ’radiofrequency ablation’ OR ’cryoablation’ OR ’mapping catheter*’ OR ’diagnostic catheter*’ OR ’cardiac electrophysiology’/exp) AND (’reus*’ OR ’reprocess*’ OR ’recycl*’ OR ’single-use’ OR ’single use’ OR ’remanufactur*’ OR ’cleaning’ OR ’disinfection’ OR ’autoclaving’ OR ’sterilisation’ OR ’sterilisation’ OR ’sterility’ OR ’decontamination’ OR ’equipment reuse’/exp) AND (’safe*’ OR ’function*’ OR ’perform*’ OR ’effective*’ OR ’efficacy’ OR ’protocol’ OR ’equivalent*’ OR ’durability’ OR ’reliability’ OR ’sterility assurance’ OR ’clinical outcomes’ OR ’risk assessment’ OR ’quality control’ OR ’infect*’ OR ’microbio*’ OR ’recommendation*’) AND [article]/lim AND [embase]/lim

### Data Synthesis

An extraction table will be created in Microsoft® Excel for Mac Version 16.85 (24051214) to standardise the extraction and analysis of the data. Collected information will include details of the first author, journal, year of publication, location, language, catheters used, safety (sterilisation method, retreatment cycles, microbial killing evidence, validation) and performance characteristics (mechanical integrity, usability).

## Reporting

### Reporting Standards

This systematic review will be reported in accordance with the Preferred Reporting Items for Systematic Reviews and Meta-Analyses (PRISMA) guidelines. A PRISMA flow diagram will be used to illustrate the study selection process, including the number of records identified, screened, deemed eligible, and ultimately included in the analysis. The resulting systematic review will be written as a research paper

### Dissemination

The completed review will be submitted for publication in a peer-reviewed journal and potentially presented at relevant cardiology or medical device conferences. Findings may also be communicated to stakeholders such as hospital management teams and regulatory bodies interested in the reprocessing of single-use medical devices.

### Transparency and Reproducibility

Any deviations from the protocol will be documented and explained in the final manuscript. All data collected will be presented in summary tables or appendices as appropriate, ensuring that other researchers can fully evaluate and replicate the review's methodology.
